# Early dental intervention in patients with X-linked hypohidrotic ectodermal dysplasia

**DOI:** 10.1186/1746-160X-8-S1-P6

**Published:** 2012-05-25

**Authors:** K Bücher

**Affiliations:** 1 Department of Conservative Dentistry, Ludwig Maximilians University, Munich, Germany

## Introduction

Familiar or syndrome-associated hypo-, oligo or anodontia in children and adolescents demands for professional dental intervention, because poor dental function and aesthetics often result in psychosocial deprivation. Treatment challenges in these patients are to develop functionally and aesthetically acceptable intermediate solutions that strive for tooth and bone preservation and allow for dental growth.

## Patients and methods

Fifteen patients with X-linked hypohidrotic ectodermal dysplasia (XL-HED) were treated at the paediatric division of our department, aiming at early intervention and oral improvement. Median age at initial presentation was 4 years. Fourteen patients showed oligodontia with 10.2 missing permanent (5.6 primary) teeth in the upper jaw, and 13 patients lacked 11.5 permanent (8.5 primary) teeth in the lower jaw. One patient had complete anodontia, another one had no teeth in the lower jaw. All oligodontic patients presented with cone-shaped teeth. None of the patients had any first permanent premolar or lateral incisor. In the lower jaw, no central incisors were present. All patients were provided with prostheses to improve masticatory function, vertical height and aesthetics. In addition three patients needed orthodontic intervention to align displaced teeth and improve aesthetical appearance. Cone-shaped teeth were modified with composites in 9 cases, one patient received veneered stainless steel crowns, and in one case ceramic crowns were inserted.

## Results and conclusion

Re-adaption of the prosthesis had to be undertaken on an individual basis according to growth. Mean time for prosthesis renewal was two years. All patients were cooperative with respect to the dental intervention. Only one patient did not accept to wear his dentures. Early intervention with conservative and prosthetic measures is a well-accepted and feasible therapeutic concept in the young patient with hypohidrotic ectodermal dysplasia.

